# Treatment of puberty trichotillomania with low-dose aripiprazole

**DOI:** 10.1186/s12991-015-0056-0

**Published:** 2015-06-17

**Authors:** Tsuyoshi Sasaki, Masaomi Iyo

**Affiliations:** Department of Child Psychiatry, Chiba University Hospital, Inohana 1-8-1, Chiba, 260-8670 Japan; Department of Psychiatry, Chiba University Graduate School of Medicine, Chiba, Japan

**Keywords:** Trichotillomania, Aripiprazole, Puberty

## Abstract

The present case is of a 14-year-old female with trichotillomania (TTM) that was treated with a low dose of aripiprazole (ARP) 1.5 mg/day. To our knowledge, this is the first published report to show an improvement of pubertal TTM using an ultra-low dose of ARP. In this case, a 50-mg fluvoxamine monotherapy for 2 years and a subsequent 4-month comprehensive cognitive behavioral therapy (CBT) monotherapy did not improve her hair-pulling symptoms. However, the treatment with a low-dose ARP of 1.5 mg/day dramatically improved her TTM symptoms without extrapyramidal symptoms. In this regard, low-dose ARP treatment for TTM might be a safe alternative to antidepressants, which carry the risk of agitation with suicidal ideation in adolescents.

## Background

Trichotillomania (TTM) is a poorly understood disorder that is characterized using DSM-5 criteria as the recurrent pulling out of one’s own hair [1]. The onset of hair pulling in TTM usually coincides with or follows the onset of puberty. TTM is associated with distress as well as with social and occupational impairment [1]. Accumulating evidence suggests that pharmacotherapy with selective serotonin reuptake inhibitors (SSRIs), tricyclic antidepressants (TCAs), antipsychotics, opioid-antagonists, or glutamate modulators has the potential to improve TTM in adults [2]. Recent empirical studies have also pointed to the potential of cognitive behavioral therapy (CBT) for the treatment of childhood TTM [3]. However, there is little information available on the treatment of TTM in puberty. In addition, caution is warranted since antidepressant therapy in children and adolescents is associated with increased rates of suicidal ideation [4–6]. Aripiprazole (ARP), a dopamine D2 receptor partial agonist, is different from other atypical antipsychotics, which usually have the profiles of D2 receptor antagonists. Moreover, ARP exhibits 5-HT2a receptor antagonism and 5-HT1a receptor partial agonism [7]. We present a pubertal patient with TTM, which was markedly improved on low-dose ARP (1.5 mg/day) after failing to respond to fluvoxamine (50 mg/day) and comprehensive CBT.

## Case presentation

A 14-year-old girl, accompanied by her mother, was seen at our hospital for treatment of trichotillomania (TTM). She had exhibited compulsive hair pulling since the age of 11. The condition had resulted in considerable hair loss, which over the preceding 3 years she had attempted to mask by wearing a wig or bandana. She had undergone several treatments for hair pulling, including school counseling and psychoeducation for her family. Most recently, she had been receiving treatment with a SSRI (fluvoxamine 50 mg/day), which was initiated at another psychiatric clinic 2 years earlier. However, her symptoms of TTM did not improve. She reported that she continued to pull her hair out despite repeated attempts to stop or decrease this activity, and the hair pulling had caused clinically significant distress or impairment in social areas. Her score on the Massachusetts General Hospital Hair Pulling Scale (MGH-HPS) [8] was 23, and she was given a diagnosis of TTM according to the DSM-5 criteria [1].

We discontinued her previous medication and started comprehensive CBT for approximately 4 months. However, her hair-pulling symptoms did not improve, and she did not wish to continue comprehensive CBT. Therefore, we discontinued the comprehensive CBT and initiated therapy with 1.5 mg/day ARP. Four weeks later, her hair-pulling symptoms were dramatically improved without extrapyramidal symptoms like sedation, weight gain, and nausea. Her MGH-HPS was reduced to six. No abnormalities were found in her general laboratory examinations. She has experienced no recrudescence of hair-pulling symptoms or extrapyramidal symptoms like sedation, weight gain, and nausea and has consistently attended school for the past 6 months.

The present case is of a 14-year-old female with TTM that was treated with a low dose of ARP 1.5 mg/day. To our knowledge, this is the first published report to show an improvement of pubertal TTM using an ultra-low dose of ARP. In this case, a 50-mg fluvoxamine monotherapy for 2 years and a subsequent 4-month CBT monotherapy did not improve her hair-pulling symptoms. However, the treatment with a low-dose ARP of 1.5 mg/day dramatically improved her TTM symptoms without extrapyramidal symptoms.

Recently, ARP has been reported to improve the TTM symptoms of adult patients [9, 10]. In addition, it has been reported that only 1.5 to 3.0 mg/day of ARP alone improved TTM symptoms in a 20-year-old man [11]. In keeping with these findings, a small (*n* = 11) open-label study suggested that ARP was a promising treatment for TTM in adults [12].

The results of a previous study [13] led to the inclusion of TTM in a new section of DSM-5, entitled Obsessive-Compulsive and Related Disorders [1]. However, TTM is not characterized by obsessional thoughts, and a greater overlap may be evident with other obsessive-compulsive spectrum disorders, such as skin picking and tic disorders [14]. A recent study showed that ARP was effective for the treatment of tic disorders in children and adolescents while causing only mild adverse effects [15]. Furthermore, Our previous report suggested that ARP might have advantages, especially in cases of a defective general status without extrapyramidal symptoms [16]. ARP may correct TTM by stabilizing dopamine in the prefrontal cortex, thereby improving motor inhibition deficits [7]. These findings suggest that the pathology of TTM may resemble that of tic disorders. In other words, TTM may be more closely aligned with addictions and disorders of habit, or tic disorders such as Tourette syndrome, than with OCD.

The main limitation of this case report was the small sample size (*n* = 1). Furthermore, fluvoxamine treatment dose of 50 mg/day might be too low for definition of SSRI resistance. It is reported that the existing trials of TTM have very small sample sizes, and the body of evidence is of low quality, mostly clinical trials where a target was more than 18 years old (only one trial included a 16 year-old-subject) [2]. So, the appropriate capacity of fluvoxamine, other SSRI, atypical, or typical antipsychotics for treatment of TTM in children and adolescents is still unknown. However, low-dose ARP treatment for TTM might be a safe alternative to antidepressants, which carry the risk of agitation with suicidal ideation in adolescents. In this regard, the mechanisms underlying the effects of low-dose ARP in the present case remain unclear, but our results do suggest that low-dose ARP may be advantageous, particularly in pubertal TTM cases like the present one.

## Conclusions

We reported the case of a female pubertal patient with TTM who was treated successfully using low-dose ARP (1.5 mg/day) monotherapy.

## Consent

Written informed consent was obtained from the patient and her mother for publication of this case report. A copy of the written consent is available for review by the Editor-in-Chief of this journal.

## Abbreviations

ARP: aripiprazole
CBT: cognitive behavioral therapy
SSRIs: selective serotonin reuptake inhibitors
TCAs: tricyclic antidepressants
TTM: trichotillomania

## References

[1] American Psychiatric Association (2013). Diagnostic and statistical manual of mental disorders.

[2] Rothbart R, Stein DJ (2014). Pharmacotherapy of trichotillomania (hair pulling disorder) an updated systematic review. Expert Opin Pharmacother.

[3] Flessner CA (2011). Cognitive-behavioral therapy for childhood repetitive behavior disorders: tic disorders and trichotillomania. Child Adolesc Psychiatr Clin N Am.

[4] Bridge JA, Iyengar S, Salary CB, Barbe RP, Birmaher B, Pincus HA (2007). Clinical response and risk for reported suicidal ideation and suicide attempts in pediatric antidepressant treatment: a meta-analysis of randomized controlled trials. JAMA.

[5] Olfson M, Marcus SC, Shaffer D (2006). Antidepressant drug therapy and suicide in severely depressed children and adults: a case-control study. Arch Gen Psychiatry.

[6] Gibbons RD, Hur K, Bhaumik DK, Mann JJ (2006). The relationship between antidepressant prescription rates and rate of early adolescent suicide. Am J Psychiatry.

[7] Pae CU, Serretti A, Patkar AA, Masand PS (2008). Aripiprazole in the treatment of depressive and anxiety disorders: a review of current evidence. CNS Drugs.

[8] O'Sullivan RL, Keuthen NJ, Hayday CF, Ricciardi JN, Buttolph ML, Jenike MA (1995). The Massachusetts General Hospital (MGH) Hairpulling Scale: 2. reliability and validity. Psychother Psychosom.

[9] Jefferys D, Burrows G (2008). Reversal of trichotillomania with aripiprazole. Depress Anxiety.

[10] Virit O, Selek S, Savas HA, Kokaçya H (2009). Improvement of restless legs syndrome and trichotillomania with aripiprazole. J Clin Pharm Ther.

[11] Yasui-Furukori N, Kaneko S (2011). The efficacy of low-dose aripiprazole treatment for trichotillomania. Clin Neuropharmacol.

[12] White MP, Koran LM (2011). Open-label trial of aripiprazole in the treatment of trichotillomania. J Clin Psychopharmacol.

[13] Grant JE, Odlaug BL, Potenza MN (2007). Addicted to hair pulling? How an alternate model of trichotillomania may improve treatment outcome. Harv Rev Psychiatry.

[14] Stein DJ, Grant JE, Franklin ME, Keuthen N, Lochner C, Singer HS (2010). Trichotillomania (hair pulling disorder), skin picking disorder, and stereotypic movement disorder: toward DSM-V. Depress Anxiety.

[15] Ho CS, Chiu NC, Tseng CF, Huang YL (2014). Clinical effectiveness of aripiprazole in short-term treatment of tic disorder in children and adolescents: a naturalistic study. Pediatr Neonatol.

[16] Sasaki T, Hashimoto T, Niitsu T, Kanahara N, Iyo M (2012). Treatment of refractory catatonic schizophrenia with low dose aripiprazole. Ann Gen Psychiatry.

